# The Second Study of Clinical and Immunological Findings in Anti-laminin 332-Type Mucous Membrane Pemphigoid Examined at Kurume University—Diagnosis Criteria Suggested by Summary of 133 Cases

**DOI:** 10.3389/fimmu.2021.771766

**Published:** 2021-11-26

**Authors:** Hua Qian, Yohei Natsuaki, Hiroshi Koga, Tamihiro Kawakami, Chiharu Tateishi, Daisuke Tsuruta, Norito Ishii, Xiaoguang Li, Takashi Hashimoto

**Affiliations:** ^1^ Central Laboratory, Dermatology Hospital of Jiangxi Province, Dermatology Institute of Jiangxi Province, and The Affiliated Dermatology Hospital of Nanchang University, Nanchang, China; ^2^ Department of Dermatology, Kurume University School of Medicine, and Kurume University Institute of Cutaneous Cell Biology, Kurume, Japan; ^3^ Department of Dermatology, Tohoku Medical and Pharmaceutical University, Sendai, Japan; ^4^ Department of Dermatology, Osaka City University Graduate School of Medicine, Osaka, Japan

**Keywords:** anti-laminin-332-type mucous membrane pemphigoid, immunoblotting, immunofluorescence, pathogenesis, diagnostic criteria

## Abstract

**Background:**

Recently, we published an article retrospectively summarizing the results in 55 anti-laminin 332 (LM332)-type mucous membrane pemphigoid (MMP) cases examined at Kurume University, which were diagnosed by strict inclusion criteria, including positive reactivity in direct immunofluorescence and absence of antibodies to non-LM332 autoantigens. However, indirect immunofluorescence using 1M-NaCl-split normal human skin (ssIIF) is also valuable for diagnosis of anti-LM332-type MMP.

**Methods:**

In this second study, we selected 133 anti-LM332-type MMP cases, which were diagnosed by our different inclusion criteria: (i) immunoglobulin G (IgG) deposition to basement membrane zone (BMZ) by direct immunofluorescence or IgG reactivity with dermal side of split skin by ssIIF, (ii) positivity for at least one of the three subunits of LM332 by immunoblotting of purified human LM332, and (iii) the presence of mucosal lesions. Clinical, histopathological, and immunological findings were summarized and analyzed statistically. Although these cases included the 55 previous cases, the more detailed study for larger scale of patients was conducted for further characterization.

**Results:**

Clinically, among the 133 patients, 89% and 43% patients had oral and ocular mucosal lesions, respectively, 71% had cutaneous lesions, and 17% had associated malignancies. Histopathologically, 93% patients showed subepidermal blisters. The sensitivities of ssIIF and direct immunofluorescence are similar but are significantly higher than indirect immunofluorescence using non-split human skin (both p < 0.001). In immunoblotting of purified LM332, patient IgG antibodies most frequently reacted with LMγ2 subunit (58%), followed by LMα3 (49%) and LMβ3 (36%). Thirty-four percent patients recognized additional non-LM332 autoantigens. Statistical analysis revealed that autoantibodies against non-LM332 autoantigens might stimulate the production of anti-LMγ2 antibodies.

**Conclusions:**

This retrospective study further characterized in more detail the clinical and immunological features of 133 cases of anti-LM332-type MMP, in which the new diagnostic criteria without positive direct immunofluorescence reactivity were useful for the diagnosis. Higher frequency with anti-LMγ2 antibodies suggested more significant pathogenic role of this subunit. Additional autoantibodies to non-LM332 autoantigens detected in one-third of the patients may contribute to complexity in anti-LM332-type MMP, including the induction of anti-LMγ2 antibodies.

## Introduction

Basal keratinocytes adhere to connective tissue at basement membrane zone (BMZ) of the epidermis (1). The interaction between keratinocytes and extracellular matrix proteins regulates many cellular behaviors, including cell adhesion, migration, proliferation, differentiation, and apoptosis (2). Laminins (LMs) are major extracellular matrices at BMZ. LMs are heterotrimeric glycoproteins consisting of three subunits, which are covalently linked by disulfide bonds, and are composed of many isoforms (1).

Laminin 332 (LM332) (previously called as epiligrin and laminin 5) is the most important LM isoform for the skin integrity (1) and is composed of α3, β3, and γ2 subunits (3). LM332 is a ligand of integrin α6β4, which is a major transmembrane component at hemidesmosome, a cell–matrix junction at BMZ (4). LM332 also adheres to integrin α3β1, which locates at focal adhesion, another adhesion device (4). LM332 is a target protein both in hereditary disease, i.e., Herlitz or non-Herlitz types of junctional epidermolysis bullosa, and in autoimmune disease, i.e., anti-LM332-type mucous membrane pemphigoid (MMP) (abbreviated as LM332-MMP in the present study) (4).

MMP (previously called as cicatricial pemphigoid) is a heterogeneous subepidermal autoimmune bullous skin disease (AIBD), which affects mainly various mucous membranes and occasionally skin (5–7). Although oral mucosa is most commonly affected, ocular, nasal, pharyngeal, laryngeal, esophageal, and genital mucosae are also involved (6). The clinical course and prognosis of MMP are affected by the specific autoantigen targeted, the titer and bioactivity profile of corresponding autoantibodies, and the specific mucosal sites of disease activity (8).

In MMP, direct immunofluorescence (DIF) and indirect immunofluorescence (IIF) tests show *in vivo* bound and circulating anti-BMZ autoantibodies of immunoglobulin G (IgG) and/or IgA subclasses, and various biochemical analyses detect a number of autoantigens (5). MMP is subdivided into two major types: anti-BP180-type MMP (BP180-MMP) and LM332-MMP. Approximately 90% and 10% of reported MMP cases are the former and the latter, respectively (1).

BP180-MMP patient show IgG and/or IgA autoantibodies reactive mainly with BP180 C-terminal domain, although LAD-1, soluble BP180 ectodomain, and BP180 NC16a domain are also occasionally recognized (5, 6). In contrast, LM332-MMP patients have IgG antibodies reactive with the 165 and 145 kDa LMα3 subunits, the 140 kDa LMβ3 subunit, and the 105 kDa LMγ2 subunit in immunoblotting (IB) of purified human LM332 (3). Recently, an IIF using recombinant LM332 was also developed for the detection of autoantibodies against LM332 in MMP sera (9).

Recent studies showed that LM332-MMP patients had an increased relative risk of cancer (7, 10, 11). However, the significance of these results is still obscure because of a limited number of patients with LM332-MMP.

Recently, we have reported a retrospective study of the clinical and immunological findings summarized for 55 LM332-MMP cases, which were diagnosed with very strict inclusion criteria, including positive DIF and the absence of other autoantigens (12). This study indicated that IIF using 1M-NaCl-split normal human skin (ssIIF) is also valuable for the diagnosis of LM332-MMP (12).

As the second version to the previous study (12), in the present study, using new inclusion criteria with positive ssIIF and concurrence of other autoantigens, we selected 133 cases of LM332-MMP from our large AIBD cohort at Kurume University, which included the 55 previous cases. Then, we further assessed in more detail both clinical features and immunological findings in the 133 cases, and extensive statistical analyses were also performed, which suggested that the new criteria are useful for the diagnosis of LM332-MMP.

## Materials and Methods

### Patients and the Information for the Clinical Features

As one of the centers for diagnosis of AIBDs in Japan, we have collected sera and information for 4,547 patients with various AIBDs, which were sent for our tests from other institutes for 14 years (2001–2014). Diagnosis of LM332-MMP was made based on our new inclusion criteria: (i) IgG deposition to BMZ by DIF or IgG reactivity with dermal side of split skin by ssIIF, (ii) positivity for at least one of the three subunits of LM332 by IB of purified human LM332, and (iii) the presence of mucosal lesions.

The information, including age, gender, medical history, and clinical features for mucocutaneous lesions, was obtained from consulting letters sent from other institutes. In addition to oral and ocular mucosal lesions, we collected information for lesions on nasal, pharyngeal, laryngeal, esophageal, and genital mucosae. Furthermore, to evaluate the severities of LM332-MMP, “oral score” (0–5) was calculated by the numbers of involved parts on oral mucosae (i.e., lip, tongue, cheek, gingiva and palate), and “mucosal score” (0–7) was calculated by the number of involved mucosae (i.e., oral, ocular, nasal, pharyngeal, laryngeal, esophageal, and genital mucosae), as we previously reported (12).

However, for some patients, information for clinical and histopathological features could not be obtained from the consulting letters. In addition, results of some serological tests could not be obtained, mainly because of the shortage of sera due to large and long-lasting nature of the present study. Therefore, assessments of most parameters were performed only for patients in whom the information of the parameters was available. This study was performed following the guidelines of Kurume University School of Medicine and Declaration of Helsinki Principles and was approved by the ethics committee of Kurume University School of Medicine.

### IF Assays

**Figure 1:**
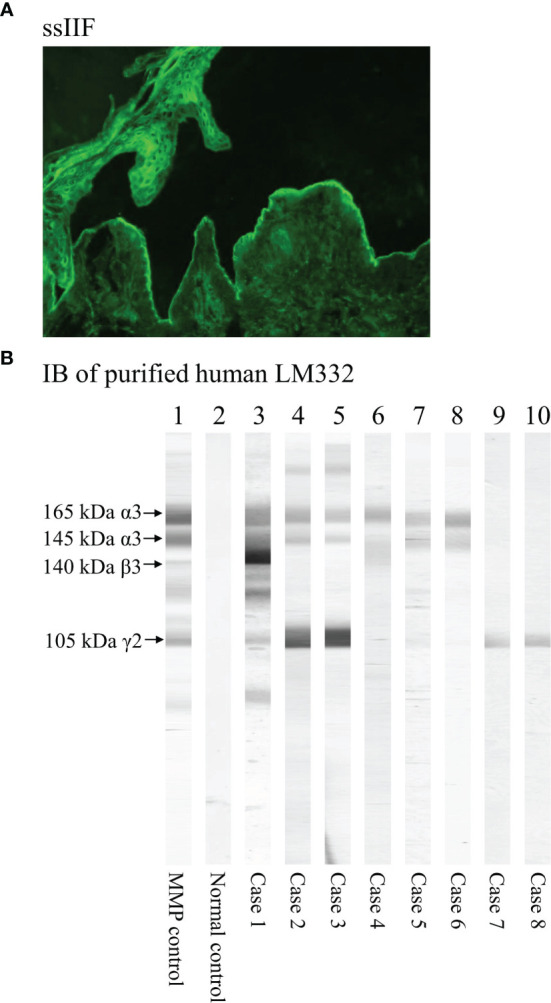
The representative figures of serological methods to detect autoantibodies in LM332-MMP. **(A)** Immunofluorescence using 1M-NaCl-split human skin (ssIIF) showing IgG reactivity with dermal side of the split by a LM332-MMP serum. **(B)** The results of immunoblotting (IB) of purified human LM332. The positive control serum (lane 1) and eight LM332-MMP sera (lanes 3–10 for cases 1–8) reacted with the three subunits of laminin-332 with various patterns, while normal control serum (lane 2) showed negative reactivity. Specifically, positive control and case 1 reacted with all the three subunits, 165 kDa and 145 kDa LMα3, 140 kDa LMβ3, and 105 kDa LMγ2 of LM332; cases 2, 3, 5, and 6 reacted with both LMα3 and LMγ2; case 4 reacted only with LMα3; and cases 7 and 8 reacted only with LMγ2.

We performed DIF using biopsy specimens from patients for depositions of IgG, IgA, IgM, and C3 to epidermal BMZ. For the diagnosis of MMP, we routinely performed two IIF assays including IIF using normal human skin and ssIIF (13–17). Normal human skin was obtained from our hospital. Both IgG and IgA autoantibodies were examined by the IIF assays. The representative result of ssIIF for a LM332-MMP case is shown in **Figure 1A**. In the result description, “DIF-not detected (ND)” indicates that DIF was not done, and therefore, the result of DIF was unknown; “DIF-” indicates that DIF was done, and the result was negative.

### IB

IB using normal human epidermal [for BP180, BP230, desmoglein 1 (Dsg1), Dsg3, envoplakin, and periplakin] and dermal extracts (for LMγ1 and collagen VII), recombinant proteins (RPs) of NC16a and C-terminal domains of BP180, concentrated culture medium of HaCaT cells, and purified human LM332 as substrates was performed as described previously (13–17). The representative results for IB of purified LM332 are shown in **Figure 1B**. “LM332 score” (1–3) was calculated by the numbers of LM332 subunits (i.e., α3, β3, and γ2 subunits) recognized by patient sera, as reported previously (12).

### ELISAs

Four commercially available ELISAs for BP180 (18), BP230 (18), Dsg1) (19), and Dsg3 (19) (MBL, Nagoya, Japan) were performed according to the protocols provided by the supplier.

### Statistical Analysis

We compared differences among various clinical parameters and immunological results with chi-square test, Fisher exact test, Mann–Whitney rank-sum test, Student’s t-test, and Pearson’s correlation by SigmaPlot 12.0 soft (Hulinks, Inc., Tokyo, Japan). p values <0.05 were considered as statistically significant.

## Results

### Diagnoses and Grouping

From the cohort of 4,547 AIBD patients, by our inclusion criteria, we diagnosed 133 patients as LM332-MMP. In the 133 cases, 55 patients (41%) showing positive IgG deposition of BMZ in DIF and exclusive reactivity with LM332, whose clinical and immunological findings had been summarized in the previously published paper (10), were defined as DIF+/LM332 group in the present study. According to the results of DIF for IgG deposition to BMZ and ssIIF for IgG reactivity with skin dermal side, the 133 cases were divided into four groups as “DIF+/ssIIF+,” “DIF+/ssIIF-,” “DIF-/ssIIF+,” and “DIF-not detected (ND)/ssIIF+”. According to the results of IIF using normal human skin for IgG reactivity to BMZ, the 133 cases were divided into two groups as “IIF+” and “IIF−”, excluding 3 cases without IIF results.

According to the autoantigens detected, the 133 cases were also divided into two groups as “sole LM332,” which reacted only with LM332, and “multiple-antigens (Ags),” which reacted with LM332 and other antigen(s).

### The Results of All the 133 LM332-MMP Patients

The 133 LM332-MMP patients consisted of 66 male and 58 female, although gender was unknown in 9 patients. The average age of patients with information for age was 66.45 years (male, 68.03 years and female, 64.60 years).

Clinically, all the 133 patients had mucosal lesions including oral (89%), ocular (43%), pharyngeal (19%), laryngeal (15%), genital (11%), nasal (6%), and esophageal (3%) lesions. A more detailed information on oral and ocular lesions is shown in **Table 1**. The average oral and mucosal scores of these 133 patients were 1.53 (SD, 0.90) and 1.88 (SD, 1.04), respectively.

**Table 1:** Clinical and immunological findings in 133 patients with anti-laminin (LM) 332-type mucous membrane pemphigoid (MMP).

Groups			All LM332−MMP (133)	DIF+/ssIIF+ (69)	DIF+/ssIIF− (17)	DIF−/ssIIF+ (12)	DIF(ND)/ssIIF+ (35)	IIF+ (73)	IIF− (57)	Sole LM332 (88)	Mutliple-Ags (45)	DIF+/LM332 (55)
**Mucosal lesions**	Oral	Erosion	92 (69%)	50 (72%)	13 (76%)	7 (58%)	22 (63%)	53 (73%)	36 (63%)	66 (75%)	26 (58%)	44 (80%)
		Blister	33 (25%)	13 (19%)	4 (24%)	5 (42%)	11 (31%)	21 (29%)	12 (21%)	17 (19%)	16 (36%)	7 (13%)
		Ulceration	11 (8%)	4 (6%)	2 (12%)	0 (0%)	5 (14%)	5 (7%)	6 (11%)	6 (7%)	5 (11%)	5 (9%)
		Unknown	6 (5%)	3 (4%)	2 (12%)	0 (0%)	1 (3%)	2 (3%)	4 (7%)	3 (3%)	3 (7%)	2 (4%)
		Total	119 (89%)	62 (90%)	15 (88%)	12 (100%)	30 (86%)	65 (89%)	51 (89%)	79 (90%)	40 (89%)	50 (91%)
	Ocular	Hyperemia	16 (12%)	10 (14%)	2 (12%)	2 (17%)	2 (6%)	12 (16%)	4 (7%)	12 (14%)	4 (9%)	7 (13%)
		Erosion	15 (11%)	9 (13%)	0 (0%)	0 (0%)	6 (17%)	12 (16%)	3 (5%)	8 (9%)	7 (16%)	5 (9%)
		Scar	17 (13%)	11 (16%)	2 (12%)	1 (8%)	3 (9%)	11 (15%)	6 (11%)	12 (14%)	5 (11%)	9 (16%)
		Unknown	18 (14%)	12 (17%)	1 (6%)	0 (0%)	5 (14%)	14 (19%)	4 (7%)	14 (16%)	4 (9%)	10 (18%)
		Total	57 (43%)	31 (45%)	5 (29%)	6 (50%)	15 (43%)	39 (53%)	18 (32%)	40 (45%)	17 (38%)	25 (45%)
	Pharyngeal		25 (19%)	12 (17%)	5 (29%)	1 (8%)	7 (20%)	15 (21%)	10 (18%)	16 (18%)	9 (20%)	11 (20%)
	Laryngeal		20 (15%)	12 (17%)	2 (12%)	3 (25%)	3 (9%)	11 (15%)	9 (16%)	13 (15%)	7 (16%)	10 (18%)
	Genital		14 (11%)	7 (10%)	2 (12%)	1 (8%)	4 (11%)	8 (11%)	6 (11%)	8 (9%)	6 (13%)	6 (11%)
	Nasal		8 (6%)	2 (3%)	0 (0%)	3 (25%)	3 (9%)	5 (7%)	3 (5%)	4 (5%)	4 (9%)	1 (2%)
	Esophageal		4 (3%)	2 (3%)	1 (6%)	1 (8%)	0 (0%)	1 (1%)	3 (5%)	3 (3%)	1 (2%)	3 (5%)
	Total		133 (100%)	69 (100%)	17 (100%)	12 (100%)	35 (100%)	73 (100%)	57 (100%)	88 (100%)	45 (100%)	55 (100%)
**Cutaneous lesions**	Lesions on trunk		75 (56%)	41 (59%)	9 (53%)	5 (42%)	20 (57%)	45 (62%)	27 (47%)	46 (52%)	29 (64%)	27 (49%)
	Lesions on limbs		77 (58%)	43 (62%)	9 (53%)	6 (50%)	19 (54%)	49 (67%)	25 (44%)	50 (57%)	27 (60%)	30 (55%)
	Blister		80 (60%)	48 (70%)	9 (53%)	5 (42%)	18 (51%)	48 (66%)	29 (51%)	52 (59%)	28 (62%)	32 (58%)
	Erythema		33 (25%)	22 (32%)	5 (29%)	3 (25%)	3 (9%)	23 (32%)	10 (18%)	19 (22%)	14 (31%)	15 (27%)
	Erosion		22 (17%)	10 (14%)	3 (18%)	3 (25%)	6 (17%)	15 (21%)	7 (12%)	13 (15%)	9 (20%)	6 (11%)
	Ulceration		5 (4%)	2 (3%)	1 (6%)	1 (8%)	1 (3%)	2 (3%)	3 (5%)	3 (3%)	2 (4%)	2 (4%)
	Total		95 (71%)	54 (78%)	10 (59%)	8 (67%)	23 (66%)	57 (78%)	35 (61%)	62 (70%)	33 (73%)	37 (67%)
**Associated malignancies**	Lung cancer		4 (3%)	3 (4%)	0 (0%)	0 (0%)	1 (3%)	1 (1%)	3 (5%)	2 (2%)	2 (4%)	2 (4%)
	Thyroid cancer		3 (2%)	1 (1%)	1 (6%)	0 (0%)	1 (3%)	2 (3%)	1 (2%)	1 (1%)	2 (4%)	1 (2%)
	Uterine cancer		2 (2%)	1 (1%)	1 (6%)	0 (0%)	0 (0%)	0 (0%)	2 (4%)	2 (2%)	0 (0%)	2 (4%)
	Tongue cancer		2 (2%)	2 (3%)	0 (0%)	0 (0%)	0 (0%)	0 (0%)	2 (4%)	2 (2%)	0 (0%)	2 (4%)
	Pancreatic cancer		2 (2%)	1 (1%)	1 (6%)	0 (0%)	0 (0%)	1 (1%)	1 (2%)	0 (0%)	2 (4%)	0 (0%)
	Prostate cancer		2 (2%)	1 (1%)	0 (0%)	1 (8%)	0 (0%)	1 (1%)	1 (2%)	2 (2%)	0 (0%)	1 (2%)
	Ovarian cancer		2 (2%)	2 (3%)	0 (0%)	0 (0%)	0 (0%)	2 (3%)	0 (0%)	1 (1%)	1 (2%)	1 (2%)
	Leukemia		2 (2%)	1 (1%)	0 (0%)	0 (0%)	1 (3%)	2 (3%)	0 (0%)	1 (1%)	1 (2%)	0 (0%)
	Gastric cancer		2 (2%)	1 (1%)	0 (0%)	0 (0%)	1 (3%)	1 (1%)	1 (2%)	2 (2%)	0 (0%)	1 (2%)
	B-cell lymphoma		2 (2%)	2 (3%)	0 (0%)	0 (0%)	0 (0%)	1 (1%)	1 (2%)	1 (1%)	1 (2%)	1 (2%)
	Adenocarcinoma		1 (1%)	0 (0%)	0 (0%)	0 (0%)	1 (3%)	1 (1%)	0 (0%)	1 (1%)	0 (0%)	0 (0%)
	Pharyngeal cancer		1 (1%)	1 (1%)	0 (0%)	0 (0%)	0 (0%)	1 (1%)	0 (0%)	1 (1%)	0 (0%)	1 (2%)
	Breast cancer		1 (1%)	0 (0%)	1 (6%)	0 (0%)	0 (0%)	0 (0%)	1 (2%)	1 (1%)	0 (0%)	1 (2%)
	Liver cancer		1 (1%)	1 (1%)	0 (0%)	0 (0%)	0 (0%)	1 (1%)	0 (0%)	0 (0%)	1 (2%)	0 (0%)
	Colon cancer		1 (1%)	0 (0%)	1 (6%)	0 (0%)	0 (0%)	1 (1%)	0 (0%)	0 (0%)	1 (2%)	0 (0%)
	Kidney cancer		1 (1%)	0 (0%)	0 (0%)	0 (0%)	1 (3%)	1 (1%)	0 (0%)	0 (0%)	1 (2%)	0 (0%)
	Total		22 (17%)	12 (17%)	4 (24%)	1 (8%)	5 (14%)	12 (16%)	10 (18%)	12 (14%)	10 (22%)	8 (15%)
**Detection methods**	DIF for BMZ (IgG)		86 in 98 (88%)	69 (100%)	17 (100%)	0 (0%)	UN	46 in 52 (88%)	40 in 46 (87%)	59 in 65 (91%)	27 in 33 (82%)	55 (100%)
	DIF for BMZ (C3)		75 in 98 (77%)	61 (88%)	14 (82%)	0 (0%)	UN	43 in 52 (83%)	32 in 46 (70%)	48 in 65 (74%)	27 in 33 (82%)	41 (75%)
	IIF for BMZ (IgG)		73 in 130 (56%)	47 (68%)	5 (29%)	6 (50%)	21 in 32 (66%)	73 (100%)	0 (0%)	45 in 85 (53%)	28 (62%)	23 (42%)
	ssIIF for reactivity with dermal side (IgG)		116 (87%)	69 (100%)	0 (0%)	12 (100%)	35 (100%)	68 (93%)	45 (79%)	81 (92%)	35 (78%)	48 (87%)
	IB of LM332 RP for 165-kDa LMα3 (IgG)		65 (49%)	37 (54%)	5 (29%)	3 (25%)	20 (57%)	37 (51%)	25 (44%)	50 (57%)	15 (33%)	32 (58%)
	IB of LM332 RP for 145-kDa LMα3 (IgG)		60 (45%)	36 (52%)	5 (29%)	1 (8%)	18 (51%)	35 (48%)	23 (40%)	46 (52%)	14 (31%)	30 (55%)
	IB of LM332 RP for LMβ3 (IgG)		48 (36%)	24 (35%)	5 (29%)	3 (25%)	16 (46%)	28 (38%)	18 (32%)	35 (40%)	13 (29%)	20 (36%)
	IB of LM332 RP for LMγ2 (IgG)		77 (58%)	41 (59%)	11 (65%)	9 (75%)	16 (46%)	44 (60%)	32 (56%)	41 (47%)	36 (80%)	28 (51%)

LM332-MMP, anti-LM332-type mucous membrane pemphigoid; DIF+/ssIIF+ group, 69 cases showing positive IgG deposition to BMZ in DIF and positive IgG staining on skin dermal side in ssIIF; DIF+/ssIIF−, 17 cases showing positive IgG deposition to BMZ in DIF but negative IgG staining on skin dermal side in ssIIF; DIF-/ssIIF+, 12 cases showing positive IgG staining on skin dermal side in ssIIF, but negative for IgG deposition to BMZ in DIF; DIF-ND (not detected)/ssIIF+, 35 cases showing positive IgG staining on skin dermal side in ssIIF, but not detected for IgG deposition to BMZ in DIF. Sole LM332 group, 88 cases positive for autoantibodies against only LM332; multiple-Ags group, 45 cases showing multiple detective autoantigens (not only LM332). DIF+/LM332 group, 55 cases showing positive IgG deposition to BMZ in DIF and only LM332 as detective autoantigen, whose clinical and immunological findings had been summarized in recently published paper (12).

In the 133 patients, 71% had cutaneous lesions with blisters as the major symptom (**Table 1**). In addition, 17% patients had associated malignancies with lung cancer as the most frequent one (**Table 1**). For various immunofluorescence detection methods, the positive rates of DIF for IgG deposition to BMZ, DIF for C3 deposition to BMZ, IIF of normal skin for IgG reactivity to BMZ, and ssIIF for IgG reactivity with dermal side were 88%, 77%, 56%, and 87%, respectively. The sensitivities of DIF for IgG deposition to BMZ and ssIIF for IgG reactivity with dermal side were similar but were significantly higher than that of DIF for C3 deposition to BMZ (both p < 0.05) and that of IIF of normal skin for IgG reactivity to BMZ (both p < 0.001). The sensitivity of DIF for C3 deposition to BMZ was also significantly higher than that of IIF of normal skin for IgG reactivity to BMZ (p < 0.05). By IB of purified LM332, the positive rates of reactivity with the 165-kDa LMα3, 145-kDa LMα3, LMβ3, and LMγ2 were 49%, 45%, 36%, and 58%, respectively. These results indicated that autoantibodies in the sera of our cohort of LM332-MMP patients most frequently reacted with LMγ2 subunit of LM332.

Histopathologically, among the 87 LM332-MMP patients available for pathological reports, 81 (93%) cases showed subepidermal blisters, with inflammatory infiltrations of eosinophils (31%), neutrophils (21%), and lymphocytes (18%) (**Table 2**).

**Table 2:** Histopathological findings in the 133 patients with anti-laminin (LM) 332-type mucous membrane pemphigoid (MMP).

Groups	All LM332-MMP (133)	DIF+/ssIIF+ (69)	DIF+/ssIIF− (17)	DIF−/ssIIF+ (12)	DIF(ND)/ssIIF+ (35)	IIF+ (73)	IIF- (57)	Sole LM332 (88)	Multiple-Ags (45)	DIF+/LM332 (55)
Subepidermal blister	81 (93%)	45 (96%)	13 (93%)	10 (91%)	13 (87%)	47 (94%)	31 (91%)	50 (94%)	31 (91%)	32 (94%)
Infiltration of eosinophils	27 (31%)	16 (34%)	6 (43%)	2 (18%)	3 (20%)	19 (38%)	8 (24%)	12 (23%)	15 (44%)	9 (26%)
Infiltration of neutrophils	18 (21%)	14 (30%)	1 (6%)	1 (9%)	2 (13%)	9 (18%)	9 (26%)	7 (13%)	11 (32%)	6 (18%)
Infiltration of lymphocytes	16 (18%)	12 (26%)	1 (6%)	1 (9%)	2 (13%)	7 (14%)	9 (26%)	11 (21%)	5 (15%)	8 (23%)
Total*	87	47	14	11	15	50	34	53	34	34

^*^The total numbers of patients who had histopathological records.

Because of the lack of detailed information for the treatments in most cases, we could not analyze the treatments.

Statistically, no clinical parameters were found to be correlated with autoantibodies against any subunits of LM332, suggesting that autoantibodies against each subunit of LM332 contribute similarly to clinical and pathological features of LM332-MMP.

### The Results of “DIF+/ssIIF+,” “DIF+/ssIIF− “DIF−/ssIIF+,” and “DIF-ND/ssIIF+” Groups

According to the results of DIF for IgG deposition to BMZ and ssIIF for IgG reactivity with dermal side, the 133 cases were divided into four groups including “DIF+/ssIIF+” (69 cases), “DIF+/ssIIF−” (17 cases), “DIF−/ssIIF+” (12 cases), and “DIF-ND/ssIIF+” (35 cases).

DIF+/ssIIF+ group showed oral lesion (90%), ocular lesion (45%), cutaneous lesion (78%), associated malignancies (17%), and positive reactivities of DIF for IgG deposition to BMZ (100%), DIF for C3 deposition to BMZ (88%), IIF of normal skin for IgG reactivity to BMZ (68%), ssIIF for IgG reactivity with dermal side (100%), and IB for the 165-kDa LMα3 (54%), the 145-kDa LMα3 (52%), LMβ3 (35%), and LMγ2 (59%) (**Table 1**). Histopathologically, among the 47 LM332-MMP patients available for pathological reports, 45 (96%) cases showed subepidermal blisters, with inflammatory infiltrations of eosinophils (34%), neutrophils (30%), and lymphocytes (26%) (**Table 2**).

The clinical, immunological, and histopathological features of DIF+/ssIIF−, DIF−/ssIIF+, and DIF(ND)/ssIIF+ groups are also summarized and shown in **Tables 1** and **2**.

### The Results of “IIF+” and “IIF−” Groups

According to the results of IIF using normal skin for IgG reactivity to BMZ, the 133 cases were divided into two groups as “IIF+” (73 cases) and “IIF−” (57 cases), excluding 3 cases without IIF results.

The IIF+ group showed oral lesion (89%), ocular lesion (53%), cutaneous lesion (78%), associated malignancies (16%), and positive reactivities of DIF for IgG deposition to BMZ (88%), DIF for C3 deposition to BMZ (83%), IIF of normal skin for IgG reactivity to BMZ (100%), ssIIF for IgG reactivity with dermal side (93%), and IB for the 165-kDa LMα3 (51%), the 145-kDa LMα3 (48%), LMβ3 (38%), and LMγ2 (60%) (**Table 1**). Histopathologically, among the 50 LM332-MMP patients available for pathological reports, 47 (94%) cases showed subepidermal blisters, with inflammatory infiltrations of eosinophils (38%), neutrophils (18%), and lymphocytes (14%) (**Table 2**).

The clinical, immunological, and histopathological features of IIF- group are also summarized and shown in **Tables 1** and **2**.

### The Results of “Sole LM332” Group and “Multiple-Ags” Group

According to the autoantigens detected, the 133 cases were divided into two groups as “sole LM332” (88 cases) and “multiple-Ags” (45 cases).

Sole LM332 group showed oral lesion (90%), ocular lesion (45%), cutaneous lesion (70%), associated malignancies (14%), and positive reactivities of DIF for IgG deposition to BMZ (91%), DIF for C3 deposition to BMZ (74%), IIF of normal skin for IgG reactivity with BMZ (53%), ssIIF for IgG reactivity with skin dermal side (92%), and IB for the 165-kDa LMα3 (57%), the 145-kDa LMα3 (52%), LMβ3 (40%), and LMγ2 (47%). Histopathologically, among the 53 LM332-MMP patients available for pathological reports, 50 (94%) cases showed subepidermal blisters, with inflammatory infiltrations of eosinophils (23%), neutrophils (13%), and lymphocytes (21%) (**Table 2**).

Multiple-Ags group showed oral lesion (89%), ocular lesion (38%), cutaneous lesion (73%), associated malignancies (22%), and positive reactivities of DIF for IgG deposition to BMZ (82%), DIF for C3 deposition to BMZ (82%), IIF of normal skin for IgG reactivity with BMZ (62%), ssIIF for IgG reactivity with skin dermal side (78%), and IB for the 165-kDa LMα3 (33%), the 145-kDa LMα3 (31%), LMβ3 (29%), and LMγ2 (80%). Histopathologically, among the 34 LM332-MMP patients available for pathological reports, 31 (91%) cases showed subepidermal blisters, with inflammatory infiltrations of eosinophils (44%), neutrophils (32%), and lymphocytes (15%) (**Table 2**).

In the 45 patients in multiple-Ags group, the numbers and rates of patients positive with various non-LM332 autoantibodies are summarized in **Table 3**. Eight different non-LM332 autoantigens were detected, with the highest positive rate (67%) for BP180 (**Table 3**). The average number of non-LM332 autoantigens recognized by patient sera was 1.6, while one patient reacted with 5 non-LM332 autoantigens, i.e., BP180, BP230, envoplakin, periplakin, and Dsg1. Thirty (67%) cases reactive with BP180 may be diagnosed as concurrence of LM332-MMP and BP180-MMP.

**Table 3:** The detailed autoantibody information of the 45 patients in multiple-Ags group.

	Autoantibodies against							
	BP180	BP230	LMγ1	Dsg3	Periplakin	Type VII collagen	Dsg1	Envoplakin
Positive patient number	30	16	7	5	4	4	4	2
Positive rate	67%	36%	16%	11%	9%	9%	9%	4%

LMγ1, laminin γ1; Dsg3, desmoglein 3; Dsg1, desmoglein 1.

In addition, among the 30 LM332-MMP cases with anti-BP180 autoantibodies, ssIIF showed IgG-positive reactivities with both skin epidermal and dermal sides in 17 patients, positive reactivity with only epidermal side in 6 patients, positive reactivity with only dermal side in 5 patients, and negative reactivity with both epidermal and dermal sides in 2 patients. The five LM332-MMP cases with anti-Dsg 3 autoantibodies and four cases with anti-Dsg 1 autoantibodies did not show the staining with keratinocyte cell surfaces in the epidermis in IIF.

### Comparative Analyses of the Results in Various LM332-MMP Groups

The results in the previously reported 55 DIF+/LM332 group patients, who showed positive IgG deposition to BMZ in DIF and reacted exclusively with LM332 (12), are also listed in **Tables 1** and **2** for comparison. We performed comparative and statistical analyses of the results in all the LM332-MMP groups shown in **Tables 1** and **2**.

Compared with the DIF+/LM332 group (55 cases), all of the LM332-MMP group (133 cases) showed no significant differences on clinical and immunological results. Compared with DIF+/LM332 group, DIF−/ssIIF+ group had higher rates on oral blister and nasal lesion (both p < 0.05) and lower positive rate of autoantibodies against the 145-kDa LMα3 in IB of purified LM332 (p < 0.05) (**Table 4**). Compared with the DIF+/LM332 group, multiple-Ags group had higher rate on oral blister (p < 0.05), accordingly lower rate on oral erosion (p < 0.05), higher positive rate of IgG reactivity to BMZ in IIF of normal skin (p < 0.05), and lower positive rates of autoantibodies against the 165-kDa LMα3 and the 145-kDa LMα3 (both p < 0.05) and higher positive rate of autoantibodies against LMγ2 (p < 0.05) in IB of purified LM332 (**Table 4**).

**Table 4:** Comparisons of DIF−/ssIIF+ and multiple-Ags groups with DIF+/LM332 group.

Groups	DIF−/ssIIF+ (12)	Multiple-Ags (45)	DIF+/LM332 (55)
Oral erosion	N.S.	26 (58%)*	44 (80%)
Oral blisters	5 (42%)*	16 (36%)*	7 (13%)
Nasal lesion	3 (25%)*	N.S.	1 (2%)
IIF for BMZ (IgG)	N.S.	28 (62%)*	23 (42%)
IB of LM332 RP for 165-kDa LMα3 (IgG)	N.S.	15 (33%)*	32 (58%)
IB of LM332 RP for 145-kDa LMα3 (IgG)	1 (8%)*	14 (31%)*	30 (55%)
IB of LM332 RP for LMγ2 (IgG)	N.S.	36 (80%)*	28 (51%)

^*^p < 0.05 when compared with DIF+/LM332 group.

N.S., not significant.

Compared with the DIF+/ssIIF+ group, the DIF+/ssIIF− group had a lower positive rate on IIF of normal skin for IgG reactivity to BMZ (p < 0.05) (**Table 5**). Compared with the DIF+/ssIIF+ group, the DIF−/ssIIF+ group had lower positive rate of autoantibody against the 145-kDa LMα3 in IB of purified LM332 (p < 0.05) (**Table 5**). Compared with the DIF+/ssIIF− group, the DIF−/ssIIF+ group showed no significant differences on clinical and immunological results.

**Table 5:** Comparisons among 4 DIF/ssIIF groups and 2 IIF groups.

Groups	DIF+/ssIIF+ (69)	DIF+/ssIIF− (17)	DIF−/ssIIF+ (12)	DIF(ND)/ssIIF+ (35)	IIF+ (73)	IIF− (57)
Ocular lesion	31 (45%)	N.S.	N.S.	N.S.	39 (53%)	18 (32%)^§^
IIF for BMZ (IgG)	47 (68%)	5 (29%)^&^	N.S.	N.S.	73 (100%)	N.S.
IB of LM332 RP for 145-kDa LMα3 (IgG)	36 (52%)	N.S.	1 (8%)^&^	N.S.	35 (48%)	N.S.

^&^p < 0.05 when compared with DIF+/ssIIF+ group.

^§^p < 0.05 when compared with IIF+ group.

N.S., not significant.

Compared with the IIF− group, the IIF+ group showed significantly higher rate of ocular lesions (p < 0.05) (**Table 5**).

Compared with the sole LM332 group, multiple-Ags group had similar statistical results to its comparison with the DIF+/LM332 group, particularly higher positive rate of autoantibodies against LMγ2 (p < 0.001) (**Table 6**). These results indicated that multiple autoantibodies might be related positively to the production of anti-LMγ2 autoantibodies, but negatively to the productions of anti-LMα3 and anti-LMβ3 autoantibodies. Compared with sole LM332 group, multiple-Ags group had higher rates of infiltration of eosinophils and neutrophils (both p < 0.05) (**Table 6**), suggesting that multiple autoantibodies might enhance the infiltration of eosinophils and neutrophils.

**Table 6:** Comparisons between sole LM332 group and multiple-Ags group.

Groups	Sole LM332 (88)	Multiple-Ags (45)
Oral erosion	66 (75%)	26 (58%)^#^
Oral blister	17 (19%)	16 (36%)^#^
ssIIF for reactivity with dermal side (IgG)	81 (92%)	35 (78%)^#^
IB of LM332 RP for 165-kDa LMα3 (IgG)	50 (57%)	15 (33%)^#^
IB of LM332 RP for 145-kDa LMα3 (IgG)	46 (52%)	14 (31%)^#^
IB of LM332 RP for LMβ3 (IgG)	35 (40%)	13 (29%)^#^
IB of LM332 RP for LMγ2 (IgG)	41 (47%)	36 (80%)^##^
Infiltration of eosinophils	12 in 53 (23%)	15 in 34 (44%)^#^
Infiltration of neutrophils	7 in 53 (13%)	11 in 34 (32%)^#^

^#^p < 0.05 when compared with sole LM332 group.

^##^p < 0.001 when compared with sole LM332 group.

In addition, oral score, mucosal score, and LM332 score were also used for statistical analyses but failed to find any correlated parameters in the present study.

## Discussion

In our previous article of the 55 LM332-MMP cases (12), we used very strict inclusion criteria of LM332-MMP: (i) IgG deposition to the BMZ in DIF, (ii) positivity to at least one of the three subunits (α3, β3, and γ2) of LM332 in IB of purified human LM332 but negative for all other known skin autoantigens, and (iii) presence of mucosal lesions. These cases are designated as DIF+/LM332 group in the present study. Based on the results of these 55 cases, we concluded that ssIIF has a diagnostic value for LM332-MMP (12). Therefore, in the present study, we used newly revised inclusion criteria of LM332-MMP, in which the diagnosis can be made on the positive reactivity with dermal side of the split skin in ssIIF in cases without positive DIF result, and cases with additional autoantibodies against non-LM332 autoantigens were also included. The new inclusion criteria enable us to obtain as many as 133 patients with LM332-MMP, including the previous 55 DIF+/LM332 group patients, although the cohort included some cases of complicated LM332-MMP with multiple autoantibodies.

In general, the 133 LM332-MMP cases in the present study showed similarly clinical and immunological features to those 55 cases in the DIF+/LM332 group, which were reported previously (12).

By comparing the DIF−/ssIIF+ group (12 cases) with DIF+/LM332 group (55 cases) (12), most clinical and pathological features showed no statistical differences, excluding oral blister and nasal lesion (both p < 0.05). This finding indicated that these two groups belong to the same subgroup of LM332-MMP and further confirmed the diagnosis values of ssIIF for LM332-MMP.

Similarly, multiple-Ags group (45 cases) and DIF+/LM332 group (55 cases) showed no statistical differences in most clinical and pathological features, also indicating that these two groups belong to the same subgroup of LM332-MMP. Therefore, patients with mucosal lesion and anti-LM332 autoantibodies could be diagnosed as LM332-MMP, regardless of the concurrence of autoantibodies to non-LM332 antigens.

Based on the results mentioned above, we propose to use our new inclusion criteria for the diagnosis of LM332-MMP, rather than the very strict diagnosis criteria used in the previous study (12).

As for diagnostic sensitivity in the present study of 133 LM332-MMP cases, sensitivity of DIF for IgG deposition to BMZ and ssIIF for IgG reactivity with dermal side were the highest, followed by DIF for C3 deposition to BMZ and IIF of normal skin for IgG reactivity to BMZ. These results implied that ssIIF is a specific and sensitive method for the LM332-MMP diagnosis. Because the result of only one of the two methods (DIF and ssIIF) may be available for some cases, we suggest to use both methods for LM332-MMP diagnosis to avoid wrong diagnosis.

In the present study, autoantibodies against LMγ2 subunit were most frequently found in LM332-MMP and may be correlated with multiple autoantibodies. These data implied that autoantibodies against LMγ2 might be different from those against LMα3 and LMβ3, in terms of pathogenic activity and possible close relation to antibodies to the other autoantigens.

Finally, in the present study, 17% of LM332-MMP patients had associated malignancies, and some patients had multiple tumors, further supporting the potential relationship between LM332-MMP and internal tumors suggested by previous reports (7, 10, 11).

In conclusion, the clinical, histopathological, and immunological features in our large cohort of LM332-MMP patients, including some complicated cases, overall confirmed the results of previous studies of LM332-MMP, including our own article (12). The diagnostic criteria for LM332-MMP, which we proposed in the present study, could benefit precise diagnosis and clinical studies on LM332-MMP in the future.

## Data Availability Statement

The original contributions presented in the study are included in the article/supplementary material. Further inquiries can be directed to the corresponding authors.

## Ethics Statement

The studies involving human participants were reviewed and approved by the ethics committee of Kurume University School of Medicine. The patients/participants provided their written informed consent to participate in this study.

## Author Contributions

HQ, XL, and TH had full access to all data in the study and took responsibility for the integrity of the data and the accuracy of the data analysis. TH and XL conceived and designed the project. HQ, YN, HK, TK, CT, DT, NI, XL, and TH collected all case information and laboratory data and analyzed the data. All authors were involved in drafting the article or revising it critically for important intellectual content, and all authors approved the final version to be published.

## Funding

This study was supported in part by Grants-in-Aid for Scientific Research (Nos. 20390308, 20591331, 21659271, 23591634, 23791298, 23791299, 23791300, 23791301, 24659534, 24591672, 24591640, and 24791185) and Supported Program for the Strategic Research Foundation at Private Universities from the Ministry of Education, Culture, Sports, Science and Technology and by “Research on Measures for Intractable Diseases” Project: matching fund subsidy from the Ministry of Health, Labour and Welfare, Japan (H24‐038), and AMED, Japan (JP19lm0203007). The study was also supported by grants from the Takeda Science Foundation and Nakatomi Foundation.

## Conflict of Interest

The authors declare that the research was conducted in the absence of any commercial or financial relationships that could be construed as a potential conflict of interest.

## Publisher’s Note

All claims expressed in this article are solely those of the authors and do not necessarily represent those of their affiliated organizations, or those of the publisher, the editors and the reviewers. Any product that may be evaluated in this article, or claim that may be made by its manufacturer, is not guaranteed or endorsed by the publisher.

## References

[1] Tsuruta D Kobayashi H Imanishi H Sugawara K Ishii M Jones JC. Laminin-332-Integrin Interaction: A Target for Cancer Therapy? Curr Med Chem (2008) 15:1968–75. doi: 10.2174/092986708785132834 PMC2992754 18691052

[2] Miner JH Yurchenco PD. Laminin Functions in Tissue Morphogenesis. Annu Rev Cell Dev Biol (2004) 20:255–84. doi: 10.1146/annurev.cellbio.20.010403.094555 15473841

[3] Burgeson RE Chiquet M Deutzmann R Ekblom P Engel J Kleinman H. A New Nomenclature for the Laminins. Matrix Biol (1994) 14:209–11. doi: 10.1016/0945-053x(94)90184-8 7921537

[4] Tsuruta D Hashimoto T Hamill KJ Jones JC. Hemidesmosomes and Focal Contact Proteins: Functions and Cross-Talk in Keratinocytes, Bullous Diseases and Wound Healing. J Dermatol Sci (2011) 62:1–7. doi: 10.1016/j.jdermsci.2011.01.005 21376539 PMC4492441

[5] Chan LS Ahmed AR Anhalt GJ Bernauer W Cooper KD Elder MJ. The First International Consensus on Mucous Membrane Pemphigoid: Definition, Diagnostic Criteria, Pathogenic Factors, Medical Treatment, and Prognostic Indicators. Arch Dermatol (2002) 138:370–9. doi: 10.1001/archderm.138.3.370 11902988

[6] Bruch-Gerharz D Hertl M Ruzicka T. Mucous Membrane Pemphigoid: Clinical Aspects, Immunopathological Features and Therapy. Eur J Dermatol (2007) 17:191–200. doi: 10.1684/ejd.2007.0148 17478379

[7] Sadler E Lazarova Z Sarasombath P Yancey KB. A Widening Perspective Regarding the Relationship Between Anti-Epiligrin Cicatricial Pemphigoid and Cancer. J Dermatol Sci (2007) 47:1–7. doi: 10.1016/j.jdermsci.2007.02.012 17467241

[8] Kourosh AS Yancey KB. Pathogenesis of Mucous Membrane Pemphigoid. Dermatol Clin (2011) 29(3):479–84. doi: 10.1016/j.det.2011.03.011. x. 21605815

[9] Goletz S Probst C Lomorowski L Schlumberger W Fechner K Beek NV. A Sensitive and Specific Assay for the Serological Diagnosis of Antilaminin 332 Mucous Membrane Pemphigoid. Br J Dermatol (2019) 180(1):149–56. doi: 10.1111/bjd.17202 30216412

[10] Egan CA Lazarova Z Darling TN Yee C Cote T Yancey KB. Anti-Epiligrin Cicatricial Pemphigoid and Relative Risk for Cancer. Lancet (2001) 357:1850–1. doi: 10.1016/S0140-6736(00)04971-0 11410196

[11] Matsushima S Horiguchi Y Honda T Fujii S Okano T Tanabe M. A Case of Anti-Epiligrin Cicatricial Pemphigoid Associated With Lung Carcinoma and Severe Laryngeal Stenosis: Review of Japanese Cases and Evaluation of Risk for Internal Malignancy. J Dermatol (2004) 31:10–5. doi: 10.1111/j.1346-8138.2004.tb00497.x 14739497

[12] Li X Qian H Natsuaki Y Koga H Kawakami T Tateishi C. Clinical and Immunological Findings in 55 Patients With Anti-Laminin 332-Type Mucous Membrane Pemphigoid. Br J Dermatol (2021) 185(2):449–51. doi: 10.1111/bjd.20099 33811327

[13] Fukuda S Tsuruta D Uchiyama M Mitsuhashi Y Kobayashi H Ishikawa T. Brunsting-Perry Type Pemphigoid With Igg Autoantibodies to Laminin-332, BP230 and Desmoplakins I/II. Br J Dermatol (2011) 165:433–5. doi: 10.1111/j.1365-2133.2011.10343.x 21457206

[14] Demitsu T Yoneda K Iida E Sasaki K Umemoto N Kakurai M. A Case of Mucous Membrane Pemphigoid With Igg Antibodies Against All the Alpha3, Beta3 and Gamma2 Subunits of Laminin-332 and BP180 C-Terminal Domain, Associated With Pancreatic Cancer. Clin Exp Dermatol (2009) 34:e992–4. doi: 10.1111/j.1365-2230.2009.03646.x 19832858

[15] Takahara M Tsuji G Ishii N Dainichi T Hashimoto T Kohno K. Mucous Membrane Pemphigoid With Antibodies to the Beta(3) Subunit of Laminin 332 in a Patient With Acute Myeloblastic Leukemia and Graft-*Versus*-Host Disease. Dermatology (2009) 219:361–4. doi: 10.1159/000243807 19797892

[16] Natsuga K Nishie W Shinkuma S Moriuchi R Shibata M Nishimura M. Circulating Iga and Ige Autoantibodies in Antilaminin-332 Mucous Membrane Pemphigoid. Br J Dermatol (2010) 162:513–7. doi: 10.1111/j.1365-2133.2009.09508.x 19751242

[17] Hashimoto T Dainichi T Ohyama B Hamada T Ishii N Sato N. A Case of Antilaminin 332 Mucous Membrane Pemphigoid Showing a Blister on the Bulbar Conjunctiva and a Unique Epitope on the Alpha3 Subunit. Br J Dermatol (2010) 162:898–9. doi: 10.1111/j.1365-2133.2010.09648.x 20199543

[18] Hayakawa T Teye K Hachiya T Uehara R Hashiguchi M Kawakami T. Clinical and Immunological Profiles of Anti-BP230-Type Bullous Pemphigoid: Restriction of Epitopes to the C-Terminal Domain of BP230, Shown by Novel Elisas of BP230-Domain Specific Recombinant Proteins. Eur J Dermatol (2016) 26(2):155–63. doi: 10.1684/ejd.2015.2719 27087683

[19] Ishii K Amagai M Hall RP Hashimoto T Takayanagi A Gamou S. Characterization of Autoantibodies in Pemphigus Using Antigen-Specific Enzyme-Linked Immunosorbent Assays With Baculovirus-Expressed Recombinant Desmogleins. J Immunol (1997) 159(4):2010–7. 9257868

